# Comparison of Point-of-Care and Laboratory Glycated Hemoglobin A1c and Its Relationship to Time-in-Range and Glucose Variability: A Real-World Study

**DOI:** 10.7759/cureus.33416

**Published:** 2023-01-05

**Authors:** Ayman Al Hayek, Wael M Alzahrani, Samia H Sobki, Abdulghani H Al-Saeed, Mohamed Al Dawish

**Affiliations:** 1 Department of Endocrinology and Diabetes, Prince Sultan Military Medical City, Riyadh, SAU; 2 Department of Clinical Chemistry, Saudi Society for Clinical Chemistry, Riyadh, SAU

**Keywords:** glucose variability, time-in-range, saudi arabia, laboratory hba1c, poct hba1c, diabetes mellitus

## Abstract

Introduction

The main objective of the current study was to perform a comparison of point-of-care testing for hemoglobin A1c (POCT-HbA1c) versus the standard laboratory method (Lab HbA1c) and their relationship to time-in-range (TIR) and glucose variability (GV) among patients with diabetes mellitus (DM) presented to the outpatient diabetes clinics.

Methods

This single-center cross-sectional study was carried out on diabetic patients (aged ≥14 years of both genders) who undergo routine follow-up at our institution and whose physicians ordered HbA1c analysis for routine care. The included patients were those using the intermittently scanned continuous glucose monitoring (isCGM) Abbott’s FreeStyle Libre system for at least three months and regular CGM users with at least 70% use.

Results

We included 97 diabetic patients (41 female and 56 male), with a median age of 25 years (Interquartile range= 18) and a mean DM duration of 10.33±5.48 years. The mean values of Lab-HbA1c and POCT HbA1c were 8.82%±0.85% and 8.53%±0.89%, respectively. The TIR, time below range, and time above range were 33.47±14.38 minutes (47.78%±14.32%), 5.44±2.58 minutes (8.41%±4.42%), and 28.8±8.27 minutes (43.81%±13.22%), respectively. According to the Bland-Altman plot analysis, the POCT-HbA1c values are consistent with the standard Lab-HbA1c values (SD of bias= 0.55, and 95% CI= -0.78 to 1.4). The univariate linear regression analysis showed a statistically significant relationship between laboratory HbA1c and POCT HbA1c (R2= 0.637, *p* <0.001), TIR (R2= 0.406, *p* <0.001), and GV (R2= 0.048, *p*= 0.032). After adjusting for age, gender, disease duration, diabetes type, and percentage of sensor data in a multivariable linear regression model, the linear associations remained significant (all* *p < 0.05).

Conclusion

The current findings show that TIR and GV can be used as endpoints and valuable parameters for the therapy of DM.

## Introduction

Diabetes mellitus (DM) affects nearly 530 million people worldwide, and its prevalence is rapidly expanding, especially in low- and middle-income countries (LMICs) [1]. Over half of all people with diabetes are undiagnosed, and only one-third have good disease control. Individuals with untreated or uncontrolled DM are more likely to develop long-term consequences and die prematurely because of cardiovascular and infectious diseases (e.g., tuberculosis and COVID-19) [2-6].

With a regional incidence of 16.2% and the second-highest forecasted rise (86%) in the number of people with DM, the Middle East and North African (MENA) area is estimated to have 136 million patients with DM by 2045 [7]. Additionally, the MENA area has the most remarkable rate of mortality from DM (24.5%) [8]. According to a WHO survey, 14.4% of Saudi Arabians were estimated to have DM [9].

Glycated hemoglobin A1c (HbA1c) is a reference metric for the circulating blood glucose concentration across the lifecycle of the RBC [10], and it has a substantial predictive value for DM-related complications [6,11,12]. In order to correctly adapt treatment, HbA1c monitoring is required. HbA1c is by far the most reliable test of long-term glycemic control, according to the WHO [13]. However, it necessitates many visits, delaying crucial therapy adjustment/intensification and decreasing long-term adherence to treatment [14]. The American Diabetes Association (ADA) guidelines of medical care advised doctors, nurses, as well as diabetes educators to employ point-of-care testing (POCT) for HbA1c in 2021 in order to help patients get timely medication modifications for improved glycemic control [11].

Time-in-range (TIR) is an appealing measurement that measures the proportion of time that an individual’s blood glucose level stays within the recommended goal range of 3.9-10.0 mmol/L or 70-180 mg/dL [15,16]. TIR is a result of the efforts of diabetes specialists to identify a reliable criterion, further than HbA1c, to evaluate glycemic control. The International consensus on TIR states that since it provides more useful information than HbA1c alone, TIR, a pivotal and emerging metric generated from CGM, has been shown to evaluate short-lived glycemic control [17]. It was determined that T1DM or T2DM patients should spend >70% (16 hours, 48 minutes) of their day within the target range, whereas elderly or high-risk T2DM individuals should spend >50% (>12 hours) [15]. Therefore, successful therapy should always aim to boost TIR while lowering time-below-range (TBR).

Debate exists about the independent impact of glycemic variability on DM-related complications that go beyond average glucose or HbA1c [18-21]. Glucose variability (GV) is troublesome, and prolonged or recurrent episodes of hypoglycemia or hyperglycemia are very concerning from a therapeutic standpoint [22]. In diabetic individuals, it is crucial to reduce GV and steer clear of spikes and troughs. Studies have shown that POCT HbA1c readings may differ from laboratory HbA1c. As a result, treatment inefficiencies may be caused by possible disparities between measured HbA1c and calculated HbA1c [23,24].

The concept of TIR and GV has challenged the traditional approach of employing HbA1c as a “one-size-fits-all” screening tool for DM care. Therefore, in this cross-sectional study, we aimed to compare POCT HbA1c and laboratory HbA1c, and their relationship to GV and TIR among patients with DM presented to the Prince Sultan Military Medical City (PSMMC). A part of the study findings was previously presented as a poster at the American Association of Clinical Endocrinology (AACE) Communities Middle East North Africa (MENA) on November 11-13, 2022 in Dubai, United Arab Emirates.

## Materials and methods

Study design

We conducted this single-center cross-sectional study at the department of endocrinology and diabetes, Diabetes Treatment Center, PSMMC, Riyadh. This study was carried out in accordance with the Helsinki Declaration. The Research and Ethics Committee of PSMMC, Riyadh, Saudi Arabia approved the study protocol (IRB approval No.# 1486). All participants gave oral and written consent after being fully briefed on the objectives and methodology of the current study.

Eligibility criteria

We included patients who fulfilled the following criteria: (a) aged 14 years and older of both genders, (b) with a diagnosis of T1D or T2D, (c) undergo routine follow-up at the outpatient diabetes center clinic (appointments of PSMMC between May and December 2020), whose physicians ordered HbA1c analysis for routine care, (d) using the intermittently scanned continuous glucose monitoring (isCGM) Abbott’s FreeStyle Libre system for at least three months, (e) regular CGM users with at least 70% use, and (f) they or their parents agreed to sign the written informed consent. We excluded (a) patients whose date of hospital POCT HbA1c and laboratory HbA1c were not corresponding to same-day visit, (b) patients who had post-bariatric surgery six weeks before Hb1Ac measure, (c) patients with diabetic ketoacidosis admission within three months prior to Hb1Ac measure, and (d) patients with diabetes duration less than one year.

Study objectives and data collection

The objectives of this study were (1) to compare same-visit POCT-HbA1c using the Cobas b 101 POC-HbA1c measurements and Lab-HbA1c, and (2) to define the relationship between TIR, GV, and HbA1c levels (POCT-HbA1c versus Lab HbA1c) as assessed by last 90 days of ambulatory glucose profile (AGP) metrics obtained reports through the LibreView web-based diabetes management system. At the screening and data collection visit, the following data were collected: (1) patient’s sociodemographic data, (2) clinical DM-related characteristics, (3) HbA1c% (Lab HbA1c and POCT-HbA1c), (4) % of time sensor is active, (5) GV, (6) TIR (70-180 mg/dL), and time above range (TAR), and TBR.

Statistical analysis

The study’s data were analyzed using the SPSS for Windows version 28. Descriptive analysis for quantitative data included mean and SD for normally distributed variables. When normal distribution was violated, the median and interquartile range were used instead of the mean and SD. For qualitative categorical variables, frequency and percentage were applied. In terms of comparative analysis for quantitative normal distributed variables, a t-test for two independent variables and ANOVA were used. Nonparametric tests such as the Mann-Whitney U and Kruskal Wallis tests were applied for not normally distributed variables. When appropriate, the Chi-square test or Exact test was applied for categorical qualitative data. The Pearson correlation coefficient (r) was used to perform the associations between the quantitative variables. Through the use of a Bland-Altman analysis, the 95% CI limits of agreement between the two HbA1c measurement techniques (POCT-HbA1c and Lab-HbA1c) in the same patients were obtained (GraphPad Prism 7.04, MD, USA).

After adjusting for relevant clinical characteristics such as patients’ age, gender, DM type, disease duration, and treatment modality, the linear regression analysis was utilized to investigate the relationship between HbA1c (dependent variable) and TIR and GV. P-value ≤ 0.05 was considered significant. The endpoints of the present study were: (a) the level of agreement between laboratory-measured and POCT-HbA1c was assessed by Pearson correlation coefficient and Bland-Altman analysis, and (b) the correlation between HbA1c (POCT-HbA1c and Lab HbA1c), TIR, and GV was assessed by the Pearson correlation coefficient.

## Results

Characteristics of the patients

Of the screened 117 patients, 97 diabetic patients were included in the current study. Most of the patients were males (n=56, 57.7%), with T1DM (n=71, 73.2%), and using multiple daily injection (MDI) insulin therapies (Table 1). 

**Table 1:** Baseline demographic and DM-related characteristics among the study population. GLP-1: Glucagon-like peptide-1; OHA: Oral antihyperglycemic agent; MDI: Multiple daily injections insulin therapy.

Characteristics		Value	
Age (Year)	Mean ± SD	29.75 ±13.55	
Gender, n (%)	Female	41	42.30%
	Male	56	57.70%
Duration of diabetes (Years)	Mean ± SD	10.33 ± 5.48	
Type of Diabetes, n (%)	Type 1 Diabetes	71	73.20%
	Type 2 Diabetes	26	26.80%
Treatment Modality, n (%)	Basal insulin plus OHA	5	5.15%
	Basal insulin plus GLP-1	4	4.12%
	Basal insulin plus OHA plus GLP-1	1	1.03%
	OHA plus MDI	4	4.12%
	MDI	56	57.73%
	MDI plus GLP-1 plus basal insulin	1	1.03%
	Premixed Insulin analogs	9	9.28%
	Premixed Insulin plus GLP-1	4	4.12%
	Insulin pump	13	13.40%

The mean age of the included patients was 29.75±13.55 years, with a mean DM duration of 10.33±5.48 years. The mean values of Lab-HbA1c and POCT HbA1c were 8.82%±0.85% and 8.53%±0.89%, respectively. Meanwhile, the mean value of % time sensor is active, and GV was 84.78±5.91 and 33.87±7.77. The TIR, TBR, and TAR were 33.47±14.38 minutes (47.78%±14.32%), 5.44±2.58 minutes (8.41%±4.42%), and 28.8±8.27 minutes (43.81%±13.22%), respectively. HbA1c and flash glucose monitoring (FGM) measures among the study population are shown in Table 2.

**Table 2:** Glycated hemoglobin A1c (HbA1c) and flash glucose monitoring (FGM) measures among the study population. POCT-HbA1c: Point-of-care testing for hemoglobin A1c; Lab-HbA1c: Laboratory glycated hemoglobin A1c.

Variable	Mean±SD
Lab-HbA1c (%)	8.82±0.85
POCT HbA1c (%)	8.53±0.89
% Time Sensor is Active	84.78±5.91
Glucose variability	33.87±7.77
Time in range	
Minute	33.47±14.38
%	47.78±14.32
Time below range	
Minute	5.44±2.58
%	8.41±4.42
Time above range	
Minute	28.8±8.27
%	43.81±13.22

Correlation analysis

**Figure 1:**
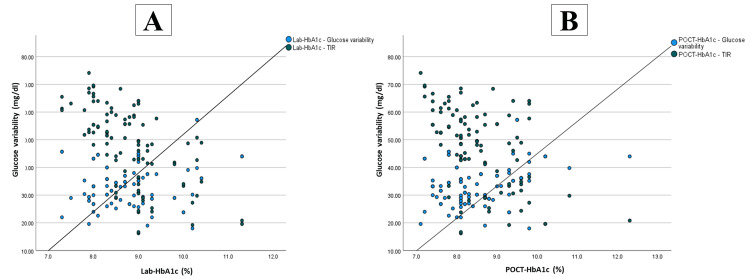
Scatter plot of the correlation between (a) Lab-HbA1c, TIR, and GV and (b) POCT-HbA1c, TIR, and GV. POCT-HbA1c: Point-of-care testing for hemoglobin A1c; Lab-HbA1c: Laboratory glycated hemoglobin A1c; TIR: Time-in-range; GV: Glucose variability.

We assessed the level of agreement between laboratory-measured and POCT HbA1c (r=0.798, 95% CI: 0.712 to 0.861). According to the Bland-Altman plot analysis, the HbA1c measurements by the POCT are in line with the standard laboratory method for HbA1c testing (Bias=0.30, SD of bias=0.55, and 95% CI: -0.78 to 1.4) (Appendix 1). As shown in Table 3 and Figure 1, we observed significant positive associations between laboratory HbA1c and TAR (p<0.001), TBR (p=0.001) and GV (p=0.032), as well as significant negative correlations between laboratory HbA1c and TIR (p<0.001), and % time sensor is active (p=0.008). Similar substantial positive associations were found between POCT-HbA1c and TAR (p<0.001), TBR (p=0.004), and GV (p=0.006), as well as significant negative correlations between POCT-HbA1c and TIR (p<0.001) and % time sensor is active (p=0.015). 

**Table 3:** The correlation between HbA1c (POCT-HbA1c versus Lab HbA1c), TIR, and GV. POCT-HbA1c: Point-of-care testing for hemoglobin A1c; Lab-HbA1c: Laboratory glycated hemoglobin A1c; TIR: Time-in-range; GV: Glucose variability; TAR: Time above range; TBR: Time below range.

	TIR	TAR	TBR	GV	% Use
Lab-HbA1c					
Correlation coefficient (r)	-0.637	0.58	0.329	0.218	-0.268
Lower Limit of 95% CI	-0.742	0.431	0.138	0.02	-0.444
Upper Limit of 95% CI	-0.502	0.699	0.496	0.4	-0.073
P-value	<0.001	<0.001	0.001	0.032	0.008
POCT-HbA1c					
Correlation coefficient (r)	-0.503	0.446	0.293	0.28	-0.247
Lower Limit of 95% CI	-0.638	0.271	0.1	0.085	-0.425
Upper Limit of 95% CI	-0.337	0.593	0.466	0.454	-0.05
P-value	<0.001	<0.001	0.004	0.006	0.015

Regression analysis

The univariate linear regression analysis showed a statistically significant relationship between laboratory HbA1c and POCT HbA1c (R2=0.637, p <0.001), TIR (R2= 0.406, p <0.001), and GV (R2= 0.048, p= 0.032). Furthermore, after adjusting for age, gender, disease duration, diabetes type, and percentage of sensor data in a multivariable linear regression model, the linear associations remained significant (all p < 0.05), as shown in Table 4.

**Table 4:** Linear and multivariate regression analyses for the association among laboratory HbA1c, POCT-HbA1c, TIR, and GV. The dependent variable is laboratory HbA1c, and the independent variables are TIR, GV, and POCT- HbA1c. Module 1 was adjusted for age and gender. Module 2 was adjusted for all variables in Model 1, plus the duration of DM and type of diabetes. Module 3 was adjusted for all variables in Model 2, plus the percentage of sensor data. POCT-HbA1c: Point-of-care testing for hemoglobin A1c; Lab-HbA1c: Laboratory glycated hemoglobin A1c; TIR: Time-in-range; GV: Glucose variability.

Model	Parameters	R	R^2^	Adj R^2^	Standardize β	B (95% CI)	P-value
Unadjusted	POCT-HbA1c	0.798	0.637	0.633	0.798	(0.645 – 0.880)	<0.001
	TIR	0.637	0.406	0.400	-0.637	(-0.047 - -0.208)	<0.001
	GV	0.218	0.048	0.038	.218	(0.002 – 0.046)	0.032
Adjusted Module 1	POCT-HbA1c	0.803	0.644	0.633	0.814	(0.658 – 0.898)	<0.001
	TIR	0.641	0.411	0.392	-0.635	(-0.047 - -0.028)	<0.001
	GV	0.231	0.054	0.023	0.219	(0.001 – 0.047)	0.045
Adjusted Module 2	POCT-HbA1c	0.806	0.650	0.639	0.808	(0.655 – 0.889)	<0.001
	TIR	0.645	0.416	0.397	-0.645	(-0.048 - -0.029)	<0.001
	GV	0.251	0.063	0.033	0.260	(0.005 – 0.051)	0.016
Adjusted Module 3	POCT-HbA1c	0.808	0.652	0.637	0.795	(0.638 – 0.881)	<0.001
	TIR	0.645	0.416	0.390	-0.652	(-0.049 - -0.028)	<0.001
	GV	0.342	0.117	0.079	0.225	(0.002 - -0.04)	0.032

## Discussion

Our study findings showed that the clinical standards of accuracy are met by the Lab-HbA1c and POCT-HbA1c agreements. In addition, we observed that a better HbA1c value (lower value) is associated with a higher percentage of TIR, less TBR, and less GV. Berbudi A et al. reported similar findings, revealing that the POCT-HbA1c and Lab-HbA1c were within the range of the agreement values. They came to the conclusion that POCT-HbA1c is a promising approach to screening and monitoring DM, particularly when a quick result is required [25]. Moreover, in non-diabetic obese patients, van Raalten F et al. showed similar results [26]. Recent experience data from PSMMC hospital demonstrated that POCT-HbA1c enhanced patient compliance with the therapist’s recommendations for the HbA1c test and increased satisfaction [27].

HbA1c may be linked to several CGM variability indicators since A1C is a weighted average of glucose exposure over the previous 2-3 months (mainly calculated from up to 2 weeks of data) [28]. Whether increased mean blood glucose is linked to significant variations will affect how glycemic control is improved. Modifications to the typical diet, medication schedule, or both are necessary for postprandial hyperglycemia treatment to be effective. For example, large meals that are high in rapidly absorbed carbohydrates and poor in dietary fiber can significantly raise postprandial blood sugar levels [29,30]. Large daily variations in blood glucose are frequently attributed to erratic eating or activity patterns and, sometimes, to skipping a pre-meal insulin bolus or administering insulin following meals. These issues should be addressed before making treatment modifications, like increasing the quantity of insulin.

Although POCT testing is not recommended to diagnose prediabetes or DM, it may offer fast access to HbA1c data at the patient visit, which is advantageous for monitoring glycemic control in healthcare settings. In addition, by improving clinical decision-making and enhancing patient-provider engagement, immediate patient input about A1C results during patient visits may improve glycemic control. An alternative, while less practical, is for diabetic patients to have blood drawn a few days before the appointment so that lab-HbA1c testing may be performed and the results are accessible during the visit [31-33].

Uncertainty exists about the impact of glucose level variations over a day or between days (based on CGM data or intermittent fingerstick blood glucose measurements). Due to the production of free radicals that have been linked to endothelial damage and the development of atherosclerotic plaques, GV has potential clinical ramifications. It has been proposed that managing short-term GV and chronic hyperglycemia may protect against microvascular and macrovascular complications [34,35]. The Diabetes Control and Complications Trial (DCCT) data analysis, however, was unable to demonstrate that intraday blood GV contributed to the emergence of microvascular complications in addition to the impact of mean blood glucose [36]. Reduced short-term GV may positively impact the onset and progression of micro- and macrovascular DM-related complications, although more evidence is still required to support this claim. In a previous study, short-term GV was linked to a higher rate of hypoglycemia [36]. Therefore, smoother glycemic control or avoiding significant spikes or falls in glucose levels would seem to be a desirable goal for all diabetic patients.

Experts in the field of DM have been working to move the emphasis away beyond HbA1c alone and toward a statistic that is more patient- and glucose-centric. As CGM has gained popularity, TIR is becoming the main indicator for evaluating DM-related complications. Previous evidence documented that individuals with T1DM and T2DM experience a 0.5% drop in HbA1c with every 10% increase in TIR [37,38]; this is in line with our findings. Beck RW et al. significantly showed that patients with T2DM receiving MDI of insulin had an improvement of their TIR to about 61% compared to 55.6% after four months of the initiation of CGM [39]. In a mixed T1DM/T2DM cohort, a meta-analysis published in 2019 found that for every 10% change in TIR, there was a 0.8% change in HbA1c. The authors concluded that %TIR has promise as a preferred measure for identifying clinical trial endpoints, estimating the likelihood of DM-related complications, and gauging a patient’s glycemic condition [37]. TIR was linked to improved clinical outcomes, even in the non-diabetic range [12]. In addition, previous studies have linked TIR to the risk factors and consequences of DM. These arguments backed the importance of % TIR as a glycemic control assessment outcome [40]. TIR has been employed in several trials to gauge glycemic control while assessing the effectiveness of various T2D management regimens. TIR was effectively used by Gal R et al. to evaluate the viability of remote CGM start in seven patients with T2DM [41]. In 124 patients with T2DM receiving MDI, the impact of liraglutide on blood sugar control was investigated in a recent trial [42]. Increased time has been spent at the “target blood glucose level,” and less time has been spent at the “very high glucose level” in the liraglutide therapy group. To treat the dawn phenomenon in patients with T2DM utilizing TIR, Zheng X et al. demonstrated the effects of moderate-intensity aerobic activity prior to breakfast. The intervention increased TIR from 83.5 ± 15.41% before exercise to 90.75 ± 12.27% after exercise [43].

A 12-week randomized controlled trial on 97 patients with uncontrolled T2DM used the changes in TIR and GV as a metric to examine the benefits of dapagliflozin and gliclazide modified release. TIR rose by about 17.4% in the group receiving gliclazide MR and 25% in the group receiving dapagliflozin. GV, as determined by the %CV, substantially improved in the gliclazide MR group by 3.8% but did not vary in the dapagliflozin group. HbA1c in neither group changed significantly from the starting point. The research identified %TIR as a potential indicator for comparing two diabetic therapeutic medications [44]. However, using POCT devices in diabetes outpatient clinic enables medical professionals to provide timely medication adjustments for improved glycemic control [14]. In adult patients with T1DM, there was a significant association between TIR and glucose management indicator (GMI). The interaction between TIR and GMI was impacted by GV, as reported by Peng HM et al. [45]. The iProTM2 sensor was used in another study that analyzed results from 91 sensors, together with demographic and clinical data, to verify the GMI formula in adults with T1DM. The link between Lab-HbA1c and GMI was influenced by GV [46]. The main drawback of the current study is the relatively small sample size from a single center (PSMMC), which may have hampered the study’s generalizability. Thus, a multicenter study with a larger sample size is warranted to confirm our findings. In addition, the research results may have been compromised by the relatively limited HbA1c range since the absence of lower HbA1c data might skew the degree of the agreement since such individuals often exhibit a wide range of glucose readings with unreported hypoglycemia episodes. Another limitation was that most of the included patients were of T1DM; thus, it is unclear if TIR and GV may be connected to T2DM risks apart from the other therapeutic goals. This should be considered in future research.

## Conclusions

The concept of TIR and GV has challenged the traditional approach of employing HbA1c as a “one-size-fits-all” screening tool for DM care. The usefulness of TIR and GV in clinical practice and clinical trials for the therapy of T1DM has been investigated. A few studies have convincingly shown the promise of TIR as a patient-centric indicator for glycemic control in patients with T2DM. Despite having similar objectives, T1DM and T2DM have different clinical/biochemical patterns and demographic predominance. The current findings show that TIR and GV can be used as endpoints and valuable parameters for the therapy of DM. Future research is necessary to get a clear picture of how TIR affects the treatment and the onset and progression of associated comorbidities.

## Figures and Tables

**Figure 2:**
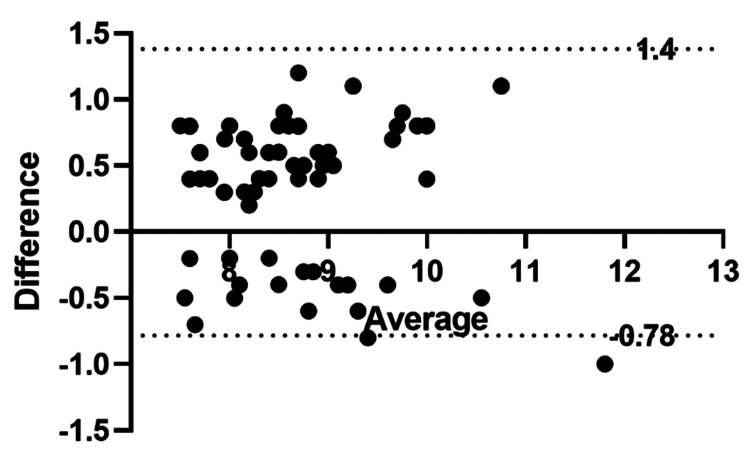
Scatter plot of the correlation between Lab-HbA1c and POCT-HbA1c. POCT-HbA1c: Point-of-care testing for hemoglobin A1c; Lab-HbA1c: Laboratory glycated hemoglobin A1c.

## Appendices

Appendix 1 
